# Correction to “First report based on the online registry of a Japanese multicenter rapid response system: A descriptive study of 35 institutions in Japan”

**DOI:** 10.1002/ams2.900

**Published:** 2023-10-11

Naito T, Fujiwara S, Kawasaki T, Sento Y, Nakada TA, Arai M, Atagi K, Fujitani S; In‐Hospital Emergency Study Group. First report based on the online registry of a Japanese multicenter rapid response system: a descriptive study of 35 institutions in Japan. Acute Med Surg. 2019; 7(1):e454.

In Table 2, the count for “Neurology” is currently listed as “1532 (26.0%)”. This is incorrect. The correct number should be “1789 (30.4%)”.

We apologize for this error.

